# Longitudinal trends in the prevalence and treatment of depression among adults with cardiovascular disease: An analysis of national health and nutrition examination survey 2009–2020

**DOI:** 10.3389/fpsyt.2022.943165

**Published:** 2022-09-08

**Authors:** Zhen Feng, Wai Kei Tong, Zhijia Tang

**Affiliations:** Department of Clinical Pharmacy and Pharmacy Administration, School of Pharmacy, Fudan University, Shanghai, China

**Keywords:** depression, cardiovascular disease, prevalence, risk factor, NHANES

## Abstract

**Objectives:**

To assess the contemporary prevalence and decadal trends of depression and antidepressant use among adults with cardiovascular disease (CVD) in the United States, as well as their risk factors from 2009 to 2020.

**Materials and methods:**

We used the National Health and Nutrition Examination Survey data to calculate the weighted prevalence of depression and antidepressant use. Adults aged 20 years or older with CVD were included. Depression and CVD were assessed by the Patient Health Questionnaire (PHQ-9) and self-report, respectively.

**Results:**

A total of 3,073 eligible participants with CVD aged >20 years were included. The overall prevalence of depression defined by PHQ-9 score ≥10 was 15.7% (95% CI 13.8–17.5), with a steady trend during 2009–March 2020 (*p* = 0.777). Female gender (aOR 1.78, 95% CI 1.20–2.64) and sleep disorder (aOR 2.62, 95% CI 1.78–3.86) were independent risk factors for depression in CVD patients, while high education level, high income, longer sleep duration, and non-current smokers were considered protective factors. The weighted prevalence of antidepressant use among depressed patients with CVD was 38.6%, which also remained unchanged during the survey period (*p* = 0.699). Participants with normal sleep pattern and duration were significantly less likely to take antidepressants (*p* = 0.003).

**Conclusion:**

The longitudinal trends in the prevalence of depression among CVD patients in the United States have been stable over the past decade, despite being significantly higher in women, and those with sleep disorders. Overall, antidepressant use was fairly low. Aggressive screening and tailored treatment are recommended for specific vulnerable subpopulations to improve their clinical outcomes.

## Introduction

Depression, also known as major depressive disorder (MDD), is one of the most common mental disorders, associated with a decline in health-related quality of life and one of the leading causes of disability (1, 2). In 2008, depression was ranked as the third leading cause of global disease burden by the World Health Organization, and is expected to rise to second place by 2030 (3). An estimated 5.0% of adults worldwide, or 280 million people, suffer from depression (4). In the United States, the prevalence of depression has risen significantly from 6.62% in 2005 to 7.28% in 2015 (2), and appears to be continuing to increase. The increase in depression has been more pronounced in recent years, from 8.7% in 2017–2018 to more than 10% in 2020 during the COVID-19 pandemic (5).

Depression is often comorbid with chronic diseases such as diabetes, hypertension, and coronary heart disease, which may further worsen health outcomes when they interact synergistically (1, 6–10). Studies have shown that 50% of people with chronic diseases suffer from depression (10). Depression can cause severe damage to personal wellbeing, occupational performance, family and social roles (11, 12). Worst of all, it can lead to suicidal behavior (13, 14). Despite the potential negative impact on individuals and society, a significant proportion of people with depression remains undertreated, especially in developing countries (4, 15–17). As the prevalence of chronic diseases rises, so does the need for effective depression management.

Previous studies have clearly suggested that patients with cardiovascular disease (CVD) experience increased risk for depression compared to the general population (15–20 vs. 5%), which makes them more vulnerable to physical limitations, low quality of life, recurrence, high mortality and high healthcare costs (18, 19). There are multiple pathophysiological mechanisms that explain the association between depression and CVD that does not occur by chance, such as increased levels of inflammatory markers, impaired heart rate variability, and hypothalamic-pituitary-adrenal (HPA) axis dysfunction (10, 20). On the other hand, depression can also affect the prognosis of existing CVD and increase its burden (21–23). CVD accompanied by depression may result in more serious health hazards than either disease alone (10, 22). As two leading causes of morbidity and mortality, a better understanding of depression prevalence and improved management in CVD patients is highly valuable from both personal and public health perspectives.

Currently, there is little evidence regarding the prevalence and treatment rates of depression in CVD patients. Although several epidemiological studies have reported relevant data, the prevalence varied widely from 11 to 65% depending on screening tools, survey samples, and disease diversity (18, 24–26). In addition, conclusions about whether characteristics such as gender and race influence the development of depression were very conflicting. Strategies such as cardiac rehabilitation, exercise, general support, cognitive behavioral therapy, and antidepressants have all been shown to be effective in depressed patients with CVD (27). Despite this, depression remains undertreated and details of antidepressant treatment were rarely reported. Therefore, this article aims to estimate the national prevalence of depression in the United States over the past decade, to investigate the relationship between CVD and depression and risk factors for depression in patients with different characteristics, and to clarify the usage patterns of antidepressants, using longitudinal data from the National Health and Nutrition Examination Survey (NHANES).

## Methods and materials

### Data sources and study populations

**FIGURE 1:**
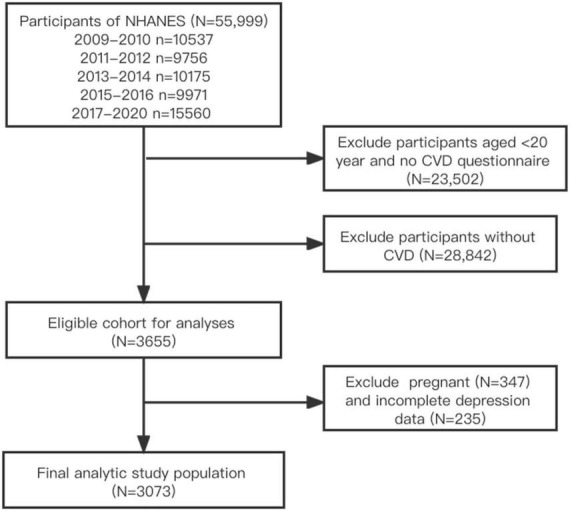
Flowchart of the screening process for the selection of eligible participants.

The NHANES is a cross-sectional survey of a nationally representative sample of the civilian, non-institutionalized United States population. Since 1999, NHANES has collected data through in-home personal interviews, as well as physical examinations and laboratory tests in mobile examination centers (MECs), and has released it publicly on a 2-year cycles (28). Accurate forecasts can often be made by combining data from different periods. The time frame of this study covered 11.2 years from 2009 to March 2020 to examine trends in the prevalence of depression among adults with CVD. Participants with CVD over 20 years of age were included, while pregnant women and those without self-reported CVD or depression were excluded. This study was exempt from Institutional Review Board review as it used de-identified, publicly available data. The inclusion and exclusion process for all eligible participants was shown in Figure 1.

### Depression assessment

Participants’ depression status was evaluated through trained interviews using the Patient Health Questionnaire (PHQ-9) in MECs. The PHQ-9 is a nine-item questionnaire that has been validated as a reliable assessment tool with good internal consistency and is recommended for screening for depression of varying severity (29, 30). The scale has a total score of 0–27, with a score ≥10 considered clinically relevant depression (CRD). This definition showed good sensitivity and specificity compared with semi and fully structured diagnostic interviews (29).

### Cardiovascular disease assessment

Cardiovascular disease was defined as any self-reported congestive heart failure, coronary heart disease, angina, heart attack or stroke. Specifically, if a participant answered “yes” to any of the questions “Has a doctor or other health professional ever told you that you have congestive heart failure/coronary heart disease/angina/heart attack/stroke?” (five separate questions), then he or she would be considered a self-reported CVD patient (31).

### Antidepressant treatment

The International Classification of Diseases, 10th Revision, Clinical Modification (ICD-10-CM)^1^ was used to refer to the reasons for receiving the treatment. On this basis, the definition of “receiving antidepressant treatment” in this study was restricted to participants’ self-reported use of antidepressant medications due to a primary diagnosis of “major depressive disorder, single episode, unspecified” (F32.9) or “major depressive disorder, recurrent, unspecified” (F33.9) (32).

### Other variables

Other variables included age, gender, race, education level, marital status, poverty income ratio (PIR), body mass index (BMI), smoking, sleep disorders, physical activity, and medical history. Marital status was categorized as cohabitated (including married and living with a partner), solitary (including widowed, divorced, or separated) and never married. Smoking status was categorized as current smoker (who has smoked 100 cigarettes in lifetime and currently smokes cigarettes), former smoker (who has smoked at least 100 cigarettes in lifetime but had quit smoking at the time of interview), and never smoker (who has never smoked or has smoked less than 100 cigarettes in lifetime) (33). Participants were also divided into three income statuses, low (PIR < 1.3), moderate (1.3 ≤ PIR < 3.5), and high (PIR ≥ 3.5), by dividing family income by a poverty threshold specific to household size and survey year, where lower PIR indicates greater poverty (34, 35). The total amount of physical activity was calculated by multiplying the number of minutes of activity per week (min/wk) by the metabolic equivalent of task (MET) of each activity, and was graded as high (MET⋅min/wk ≥ 600) and low (MET⋅min/wk < 600) (36). Regarding the criteria for the presence of comorbidities, diabetes was defined as self-reported physician-diagnosed diabetes, current use of hypoglycemic medications, fasting plasma glucose level ≥126 mg/dL, HbA1c ≥6.5% (37), or 2-h blood glucose (OGTT) ≥200 mg/dL. Hypertension was defined as systolic blood pressure ≥130 mmHg, diastolic blood pressure ≥80 mmHg, self-reported hypertension, or taking antihypertensive medications, where SBP and DBP were obtained by calculating the average of all available blood pressure measurements (37). Hypercholesterolemia was defined as self-reported total cholesterol level ≥240 mg/dL, or taking lipid-lowering drugs. Chronic kidney disease (CKD) was defined as a self-reported diagnosis of CKD.

### Study goals and objectives

The primary objectives of this study were to investigate the current prevalence and decadal trends of depression, and to identify predictors of depression among adults with CVD; a secondary objective was to determine the proportion of adults with depression and CVD who are treated with antidepressants.

### Statistical analysis

In accordance with the National Center for Health Statistics analysis guidelines, data were weighted to ensure that appropriate estimates were representative of the total civilian non-institutionalized United States population (37–39). Continuous variables were expressed as weighted mean [standard error (SE)], and categorical variables were expressed as number (weighted percentage). The Rao-Scott χ^2^ test was used to assess whether there were significant differences in sociodemographic characteristics between the depression and non-depression groups. The Taylor linearization method was used to estimate SEs and 95% confidence intervals (CI). Survey-weighted multiple logistic regression analysis was used to identify predictors of depression among CVD patients by calculating odds ratios and 95% CIs, with all categorical variables described above as covariates. To examine trends from 2009 to 2020, we reported *p*-values for logistic regression with the year of NHANES as a continuous variable (ordered categorical) predicting the prevalence of depression. A 2-sided *p* < 0.05 was considered statistically significant. Data analyses were performed using R Studio (version 1.4.1717, PBC, United States).

## Results

### Characteristics of the analytic sample

A total of 3,073 participants were analyzed for this study, representing a weighted total population of 18,925,324. Of the 534 participants with depression, the majority (62.7%) were female, 52.8% were older than 60, and 66.5% were non-Hispanic whites (Table 1). Significant differences were found between the depression and non-depression group in age, gender, race, education level, BMI, PIR, marital status, smoking, sleeping duration, and presence of sleep disorder and CKD (*p* < 0.05).

**TABLE 1:** Sociodemographic and clinical characteristics of participants in NAHNES 2009–March 2020.

Characteristics	No. of participants (weighted %)			*P*-value
	
	Total	Non-depression	Depression	
Overall	3,073 (100)	2,539 (84.3)	534 (15.7)	
**Age, year**				
20–39	114 (4.5)	82 (3.8)	32 (7.9)	<0.001
40–59	657 (24.5)	485 (21.7)	172 (39.3)	
≥60	2,302 (71.1)	1,972 (74.5)	330 (52.8)	
**Gender**				
Male	1,764 (54.6)	1,539 (57.8)	225 (37.3)	<0.001
Female	1,309 (45.4)	1,000 (42.2)	309 (62.7)	
**Race**				
Non-Hispanic white	1,563 (73.4)	1,324 (74.7)	239 (66.5)	<0.001
Mexican American	272 (4.3)	203 (3.8)	69 (6.9)	
Non-Hispanic black	736 (11.5)	610 (10.8)	126 (14.9)	
Other	502 (10.8)	402 (10.6)	100 (11.6)	
**Education level**				
Secondary or below	897 (20.6)	684 (18.8)	213 (30.3)	<0.001
High school/associate	1,700 (59.7)	1,408 (58.8)	292 (64.4)	
College graduate or above	473 (19.8)	444 (22.4)	29 (5.4)	
**BMI, kg/m^2^**				
<25	618 (19.6)	510 (19.6)	108 (19.6)	<0.001
25–29.9	942 (30.9)	815 (32.9)	127 (19.8)	
≥30	1,425 (49.5)	1,148 (47.5)	277 (60.6)	
**PIR**				
≤1.3	1,058 (26.7)	796 (23.4)	262 (44.3)	<0.001
1.31–3.5	1,125 (42.3)	958 (42.3)	167 (42.5)	
>3.5	598 (31.0)	551 (34.3)	47 (13.2)	
**Marital status**				
Cohabitated	1,640 (59.0)	1,427 (61.3)	213 (46.4)	<0.001
Solitary	1,179 (33.5)	930 (32.3)	249 (39.7)	
Never married	252 (7.6)	181 (6.4)	71 (14.0)	
**Smoking**				
Current smoker	684 (21.7)	505 (18.7)	179 (37.7)	<0.001
Former smoker	1,181 (39.0)	1,018 (40.9)	163 (28.3)	
Never smoker	1,205 (39.4)	1,014 (40.3)	191 (34.1)	
**Sleep disorder**	1,333 (44.7)	974 (39.8)	359 (71.2)	<0.001
**Sleeping duration**				
<6 h	515 (14.6)	371 (12.2)	144 (27.6)	<0.001
6–8 h	1,809 (62.0)	1,554 (64.2)	255 (50.1)	
>8 h	720 (23.4)	594 (23.6)	126 (22.3)	
**Physical activity**				
Low	1,015 (53.4)	855 (52.3)	160 (60.8)	0.082
High	773 (46.6)	677 (47.7)	96 (39.2)	
Diabetes	1,219 (36.3)	974 (34.8)	245 (44.3)	0.696
Hypertension	2,588 (81.4)	2,143 (81.7)	445 (79.9)	0.549
Hypercholesterolemia	2,221 (74.5)	1,816 (73.9)	405 (77.9)	0.296
CKD	378 (9.8)	293 (9.0)	85 (14.2)	0.010

BMI, body mass index; PIR, poverty income ratio; CKD, chronic kidney disease.

### Prevalence and risk factors of depression

From 2009 to March 2020, the estimated prevalence of depression (defined by PHQ-9 score ≥ 10) among adults with CVD in the United States was 15.7% (95% CI 13.8–17.5%), equivalent to 2.96 million adults. As shown in Table 2, female gender (aOR 1.78, 95% CI 1.20–2.64), and sleep disorder (aOR 2.62, 95% CI 1.78–3.86) remained significant as an independent predictor of depression in adults with CVD after controlling for covariates. In contrast, patients aged ≥60 years, those with college graduates or above, PIR ≥3.5, non-current smoker, and sleeping duration ≥6 h per night were shown to decrease risk of depression.

**TABLE 2:** Prevalence and odds ratios for depression by characteristics among adults with cardiovascular disease in the United States, 2009–March 2020.

Characteristics	Total prevalence,% (95% CI)	Odds ratio (95% CI)	
		
		Unadjusted	Adjusted
**Age, years**			
20–39	27.5 (17.3–37.8)	1.00 (reference)	1.00 (reference)
40–59	25.2 (20.3–30.1)	0.89 (0.50–1.56)	0.84 (0.43–1.65)
≥60	11.6 (9.8–13.4)	0.35 (0.20–0.61)	0.48 (0.25–0.91)
**Gender**			
Male	10.7 (8.7–12.7)	1.00 (reference)	1.00 (reference)
Female	21.6 (18.7–24.6)	2.31 (1.76–3.03)	1.78 (1.20–2.64)
**Race**			
Non-Hispanic white	14.2 (11.9–16.4)	1.00 (reference)	1.00 (reference)
Mexican American	25.2 (20.9–29.6)	2.04 (1.52–2.75)	1.18 (0.71–1.97)
Non-Hispanic black	20.3 (16.9–23.7)	1.55 (1.16–2.06)	0.64 (0.41–0.98)
Other	16.9 (13.2–20.6)	1.23 (0.92–1.65)	0.97 (0.68–1.39)
**Education level**			
Secondary or below	23.0 (19.4–26.7)	1.00 (reference)	1.00 (reference)
High school/associate	16.9 (14.4–19.3)	0.68 (0.52–0.89)	0.67 (0.47–0.98)
College graduate or above	4.3 (2.1–6.4)	0.15 (0.09–0.26)	0.28 (0.13–0.57)
**BMI, kg/m^2^**			
<25	15.5 (11.8–19.1)	1.00 (reference)	1.00 (reference)
25–29.9	9.9 (7.7–12.2)	0.60 (0.43–0.85)	0.64 (0.39–1.05)
≥30	18.9 (16.0–21.9)	1.28 (0.88–1.84)	1.42 (0.84–2.40)
**PIR**			
≤1.3	26.0 (22.6–29.4)	1.00 (reference)	1.00 (reference)
1.31–3.5	15.7 (13.0–18.4)	0.53 (0.41–0.69)	0.73 (0.51–1.04)
≥3.5	6.6 (3.9–9.5)	0.20 (0.12–0.33)	0.40 (0.23–0.70)
**Marital status**			
Cohabitated	12.3 (10.0–14.6)	1.00 (reference)	1.00 (reference)
Solitary	18.5 (15.2–21.9)	1.62 (1.18–2.21)	1.02 (0.63–1.65)
Never married	28.9 (20.6–37.1)	2.89 (1.80–4.66)	1.85 (0.99–3.47)
**Smoking**			
Current smoker	27.2 (22.6–31.8)	1.00 (reference)	1.00 (reference)
Former smoker	11.4 (8.5–14.2)	0.34 (0.24–0.48)	0.54 (0.30–0.97)
Never smoker	13.5 (11.3–15.8)	0.42 (0.32–0.55)	0.57 (0.37–0.86)
**Sleep disorder**			
No	8.1 (6.3–10.0)	1.00 (reference)	1.00 (reference)
Yes	25.0 (21.9–28.0)	3.75 (2.82–4.99)	2.62 (1.78–3.86)
**Sleeping duration**			
<6 h	29.3 (24.3–34.4)	1.00 (reference)	1.00 (reference)
6–8 h	12.6 (10.4–14.8)	0.35 (0.26–0.46)	0.49 (0.29–0.85)
>8 h	14.8 (11.7–18.0)	0.42 (0.29–0.60)	0.54 (0.34–0.84)
**Physical activity**			
Low	15.0 (12.1–18.0)	1.00 (reference)	1.00 (reference)
High	11.1 (7.7–14.4)	0.71 (0.47–1.05)	0.70 (0.43–1.12)
**Diabetes**			
No	24.5 (–5.0 to 54.0)	1.00 (reference)	1.00 (reference)
Yes	19.1 (15.3–22.9)	0.73 (0.14–3.67)	0.35 (0.09–1.42)
**Hypertension**			
No	15.3 (11.8–18.8)	1.00 (reference)	1.00 (reference)
Yes	15.4 (13.4–17.3)	1.06 (0.77–1.47)	0.85 (0.60–1.26)
**Hypercholesterolemia**			
No	14.0 (10.0–18.0)	1.00 (reference)	1.00 (reference)
Yes	16.4 (14.4–18.4)	1.20 (0.85–1.67)	1.51 (0.97–2.36)
**CKD**			
No	16.4 (14.4–18.4)	1.00 (reference)	1.00 (reference)
Yes	22.7 (16.9–28.5)	1.68 (1.13–2.49)	1.34 (0.75–2.39)

### Temporal trends in the prevalence of depression during the study period (2009–March 2020)

**FIGURE 2:**
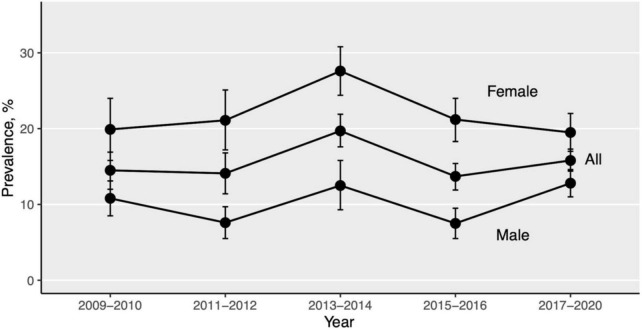
Trends in prevalence of depression by gender, 2009–March 2020. Error bars indicate standard errors. Specific estimates are shown in Table 3.

As demonstrated in Figure 2 and Table 3, the prevalence of depression (defined by PHQ-9 score ≥ 10) did not change significantly from 2009 to March 2020, neither by sociodemographic characteristics nor by comorbidities (*p* > 0.05), except in the high-level physical activity group (*p* = 0.025). Nevertheless, the year 2013–2014 witnessed the highest prevalence at 19.7% (95% CI 15.1–24.4), and regardless of the survey period, women were more likely to suffer from depression than men.

**TABLE 3:** Trends in the prevalence of depression among adults with cardiovascular disease in the United States, 2009–March 2020.

	Prevalence, % (95% CI)					P for trend
	
	2009–2010 (*n* = 88)	2011–2012 (*n* = 85)	2013–2014 (*n* = 107)	2015–2016 (*n* = 83)	2017–2020 (*n* = 171)	
Overall	14.5 (9.2–19.7)	14.1 (8.3–19.9)	19.7 (15.1–24.4)	13.7 (10.0–17.4)	15.8 (12.9–18.8)	0.777
Age, years*						
<60	26.4 (13.4–39.4)	21.4 (11.2–31.6)	29.2 (15.4–43.0)	23.9 (14.0–33.8)	26.2 (18.3–34.1)	0.867
≥60	8.5 (5.4–11.6)	11.0 (5.2–16.8)	16.1 (9.6–22.6)	9.9 (6.4–13.4)	11.8 (9.3–14.3)	0.506
**Gender**						
Male	10.8 (5.9–15.7)	7.6 (3.2–12.0)	12.5 (5.6–19.5)	7.5 (3.2–11.8)	12.8 (9.1–16.4)	0.354
Female	19.9 (11.2–28.6)	21.1 (12.8–29.5)	27.6 (20.8–34.4)	21.2 (15.1–27.2)	19.5 (14.4–24.7)	0.648
**Race**						
Non–Hispanic white	11.4 (5.3–17.5)	13.1 (6.3–20.0)	17.4 (11.5–23.2)	13.7 (7.9–19.5)	14.5 (11.0–17.9)	0.493
Mexican American	29.7 (21.6–37.7)	17.6 (9.0–26.3)	33.5 (17.5–49.4)	16.7 (10.5–22.9)	26.4 (18.5–34.2)	0.662
Non-Hispanic black	22.8 (14.8–30.8)	19.0 (11.8–26.2)	25.6 (16.3–34.8)	14.9 (7.0–22.8)	20.7 (13.9–27.5)	0.574
Other	22.7 (4.4–41.1)	13.6 (5.5–21.6)	25.5 (11.8–39.3)	11.6 (7.5–15.6)	17.2 (11.1–23.4)	0.602
Education level*						
Secondary or below	24.0 (17.2–30.7)	19.8 (12.0–27.6)	27.5 (17.1–37.9)	23.9 (12.4–35.4)	21.4 (13.6–29.2)	0.860
High school or above	10.6 (5.9–15.4)	12.2 (6.4–18.0)	17.8 (11.5–24.1)	11.4 (7.2–15.6)	14.8 (11.3–18.2)	0.329
**BMI**						
<25	17.1 (7.9–26.3)	10.0 (2.2–17.8)	13.8 (3.5–24.2)	18.5 (7.5–29.5)	17.1 (11.0–23.3)	0.406
25–29.9	10.7 (5.3–16.3)	7.4 (0.8–13.9)	12.9 (8.4–17.4)	8.2 (3.5–13.0)	9.9 (5.4–14.5)	0.947
≥30	16.0 (8.3–23.6)	19.1 (11.4–26.9)	27.0 (16.2–37.9)	14.8 (9.7–19.9)	18.6 (13.7–23.4)	0.945
PIR*						
≤1.3	30.2 (22.9–37.4)	27.1 (16.6–37.6)	22.3 (17.6–27.0)	23.1 (16.6–29.6)	27.9 (19.8–36.1)	0.734
>1.3	10.2 (3.7–16.7)	8.7 (3.9–13.6)	22.3 (17.6–27.0)	9.8 (4.3–15.3)	12.2 (9.5–14.9)	0.571
**Marital status**						
Cohabitated	12.5 (8.2–16.8)	8.1 (2.4–13.8)	18.7 (11.6–25.9)	10.9 (6.3–15.6)	11.7 (7.5–15.8)	0.860
Solitary	15.2 (5.8–24.6)	22.9 (15.0–30.9)	20.2 (13.4–27.0)	15.3 (9.6–21.0)	18.7 (11.3–26.2)	0.404
Never married	32.5 (19.8–45.1)	23.4 (4.1–42.6)	25.5 (8.5–42.6)	23.7 (5.2–42.1)	36.1 (19.2–52.9)	0.640
**Smoking**						
Current smoker	26.9 (14.3–39.6)	19.2 (5.6–32.7)	29.9 (19.3–40.6)	25.2 (16.7–33.7)	32.0 (23.7–40.3)	0.254
Former smoker	8.0 (4.6–11.3)	9.4 (2.8–16.0)	17.3 (6.4–28.3)	8.8 (1.2–16.3)	12.0 (7.5–16.6)	0.415
Never smoker	14.6 (8.2–21.1)	15.5 (10.3–20.8)	16.5 (9.7–23.3)	10.3 (5.3–15.2)	12.1 (8.2–16.0)	0.196
**Sleep disorder**	23.6 (14.6–32.5)	23.8 (15.4–32.2)	30.7 (20.4–40.9)	21.8 (13.9–29.6)	25.0 (21.1–29.0)	0.986
**Sleeping duration**						
<6 h	30.0 (19.8–40.2)	35.2 (22.8–47.6)	27.3 (13.6–40.9)	25.4 (10.7–40.2)	28.4 (17.9–38.9)	0.509
6–8 h	9.6 (5.6–13.6)	9.3 (4.4–14.2)	19.9 (5.5–15.4)	11.2 (6.5–15.8)	12.1 (7.2–17.0)	0.481
>8 h	15.2 (2.3–28.1)	13.2 (1.4–24.9)	10.4 (14.6–25.1)	13.4 (6.6–20.2)	16.6 (11.9–21.3)	0.455
**Physical activity**						
Low	17.1 (7.5–26.7)	10.8 (2.4–19.3)	18.3 (9.1–27.5)	13.7 (8.7–18.8)	14.9 (10.5–19.4)	0.902
High	6.2 (1.6–10.8)	5.0 (0.8–9.3)	12.4 (3.7–21.1)	12.7 (3.3–22.1)	13.5 (7.4–19.7)	0.025
Diabetes	18.8 (11.7–26.0)	23.5 (12.8–34.2)	22.4 (12.7–32.0)	14.5 (6.7–22.4)	18.0 (10.8–25.3)	0.379
Hypertension	13.8 (8.7–18.9)	13.9 (8.0–19.7)	18.7 (12.5–25.0)	12.7 (9.8–15.5)	16.4 (12.8–20.0)	0.504
Hypercholesterolemia	15.5 (8.4–22.5)	15.8 (9.9–21.7)	18.6 (13.0–24.1)	14.8 (10.8–18.7)	16.7 (13.4–19.9)	0.879
CKD	19.2 (9.5–28.8)	27.0 (9.2–44.8)	23.0 (10.4–35.6)	21.5 (0.1–42.9)	22.6 (13.3–31.9)	0.988

*Subgroups with insufficient sample size were pooled.

### Antidepressant treatment in cardiovascular disease patients with depression

The weighted prevalence of antidepressant use among CVD patients with depression was 38.6% (95% CI 30.8–46.5%) from 2013 to March 2020, and remained essentially unchanged during the survey period (*p* for trend = 0.699). Of the patients who reported taking antidepressants, 42.4% were using selective serotonin reuptake inhibitors (SSRIs). We observed that people without sleep disorders and those who slept 6–8 h a day were less likely to use antidepressants than their counterparts (*p* = 0.003). There were no statistically significant differences between other subgroups (Table 4).

**TABLE 4:** Antidepressant use among depressed cardiovascular patients in the United States, 2013–March 2020.

Characteristics	Prevalence, weighted % (95% CI)	*P*-value
Overall	38.6 (30.8–46.5)	
Age, years*		
<60	44.2 (31.5–56.9)	0.146
≥60	34.1 (25.9–42.2)	
**Gender**		
Male	34.9 (23.7–46.1)	0.458
Female	40.9 (30.0–51.8)	
**Race**		
Non-Hispanic white	42.9 (33.2–52.7)	0.291
Mexican American	17.5 (5.9–29.1)	0.225
Non-Hispanic black	32.6 (16.5–48.6)	0.989
Other	32.7 (15.2–50.2)	Reference
Education level*		
Secondary or below	35.2 (23.8–46.5)	0.560
High school or above	39.9 (29.6–50.1)	
**BMI**		
<25	41.7 (24.1–59.3)	0.473
25–29.9	48.4 (32.8–63.9)	0.133
≥30	34.6 (24.1–45.2)	Reference
PIR*		
≤1.3	40.4 (30.1–50.7)	0.654
>1.3	37.2 (25.7–48.6)	
**Marital status**		
Cohabitated	37.8 (25.0–50.6)	0.597
Solitary	37.4 (26.7–48.1)	0.571
Never married	44.1 (23.5–64.7)	Reference
**Smoking**		
Current smoker	45.6 (32.7–58.5)	0.162
Former smoker	34.0 (23.3–44.7)	0.940
Never smoker	34.6 (21.9–47.4)	Reference
**Sleep disorder**		
Yes	45.0 (35.6–54.5)	0.003
No	21.1 (10.0–32.2)	
**Sleeping duration**		
<6 h	40.0 (21.9–58.0)	0.190
6–8 h	30.1 (20.7–39.6)	0.003
>8 h	55.2 (43.0–67.3)	Reference
**Physical activity**		
Low	38.7 (23.5–53.9)	0.792
High	36.1 (23.9–48.2)	

*Subgroups with insufficient sample size were pooled.

## Discussion

In this nationally representative sample of United States adults aged 20 years and older, we observed that the prevalence of depression (defined by PHQ-9 score ≥ 10) among self-reported CVD patients remained fairly high, despite the trend plateauing over the past decade (*p* for trend = 0.777). Specifically, the estimated prevalence was 15.7% (95% CI 13.8–17.5%) during 2009–March 2020, which was consistent with the prevalence of 15–20% reported in previous studies (21, 40).

In this study, we found that women were almost twice as likely to be depressed as men (21.6 vs. 10.7%) and had a significantly higher risk (aOR 1.78, 95% CI 1.20–2.64), even after controlling for other variables. Similar findings have been documented in other studies conducted in patients with myocardial infarction (MI) (41). The lifetime prevalence of depression and the occurrence of depressive symptoms at the onset of acute MI were also markedly higher in women than in men (48 vs. 24%, 39 vs. 22%, respectively; *p* < 0.0001) (42, 43). This phenomenon could be partly explained by the fact that women were indeed more prone to depression than men, and not just because they were more likely to remember or report criterion symptoms (44).

Besides this, we noticed that the prevalence of depression was lowest among people aged ≥60 years (11.6%) and non-Hispanic whites (14.2%). Our findings were consistent with previous literature on age (45), but not on racial differences (46, 47), probably due to the use of different screening tools and diagnostic criteria. In our final regression model, blacks are associated lower risk of depression than in whites. Consistently, previous research has found that whites have higher rates of depression than most other racial groups (2). Johnson et al. (46) and Pugh et al. (47) even explicitly claimed that white race was a risk factor for post-stroke depression. Further research is warranted to verify the effect of race on depression among patients with CVD.

In terms of marital status, the unmarried were more likely to suffer from depression than those who were or used to be married despite the association was not significant in the final model. Previous research has repeatedly shown that marital status was associated with depression, and that being married had a protective effect (48–51). These findings prompt clinicians to pay special attention when screening for depression in such subpopulations and further studies are needed to clarify these relations.

Moreover, sleep disorder, another risk factor, nearly tripled the risk of depression in patients with CVD (aOR 2.62, 95% CI 1.78–3.86). Evidence suggested sleep disorders put people at risk for CVD and depression (52). We found an inverse relationship between sleep duration and depression, with those who slept 6–8 h and >8 h per night having a significantly lower risk of depression compared to those who slept <6 h (aOR 0.49, 95% CI 0.29–0.85; 0.54, 95% CI 0.34–0.84, respectively), which was consistent with the literature (53, 54). Although the association between sleep deprivation and depression has been largely established, the impact of prolonged sleep on depression remained controversial (53, 55). In line with a study that included general adults in the NHANES from 2009 to 2016 (55), our findings suggested that those with 6–8 h of sleep had the lowest prevalence (12.6%), not only sleep deprivation but also excessive sleep was associated with depression. Further studies are warranted to clarify the possible causal relationship and underlying mechanisms between sleep and depression.

Early and timely identification through aggressive screening and tailored treatment are recommended when dealing with those vulnerable populations in clinical practice. Correspondingly, high-educated, overweight, high-income, and non-smoking patients with CVD had a lower risk of depression, but still cannot be ignored.

Despite the potential for adverse outcomes, some people with depression, such as women, were undertreated (56). In this study, antidepressant use among United States CVD patients remained steady during 2013–2020 (*p* for trend = 0.699), with only 38.6% of depressed patients reporting receiving antidepressant therapy. Those with normal sleep patterns and sleep duration were significantly less likely to take the medication (*p* = 0.003). Among all antidepressants, SSRIs were the most popular choice (42.4%). SSRIs were also considered a relatively safe treatment option which did not increase the rate of cardiovascular side effects or cardiac events in patients with coronary heart disease compared with placebo, and showed modest positive treatment effects (57–60). In contrast, the 2021 European Society of Cardiology (ESC) guidelines have raised specific concerns about increased risk of sudden cardiac death with psychopharmacological treatment (61). Traditional antidepressants, such as tricyclic antidepressants, may increase the risk of cardiac events, all-cause mortality, and adverse drug interactions (62). Rational use of antidepressants in the setting of CVD remains challenging given the current state of the evidence. Further studies are needed to evaluate the safety of other classes of antidepressants. Moreover, cognitive behavioral therapy (CBT), psychotherapy (or “talk therapy”) and nurse-led support can also facilitate patients at risk for CVD (63, 64). Given that depression is strongly related to CVD development and poorer prognosis (10, 61), prompt diagnosis and effective management strategies for depression are critical.

This study has several limitations. First, while we have quantified the disease burden in terms of prevalence and determined risk factors for depression, it should be emphasized that cross-sectional data cannot confirm causality. In addition, quality of life, disease-related costs and potential complications associated with depression were not analyzed. Second, although our study covered a large population sample, it may be subject to response bias and sampling bias due to the use of self-reported PHQ-9, CVD diagnoses, and antidepressant treatment, underestimating target participants, and nor is it absolutely equivalent to a clinical diagnosis of the disease. Therefore, the accuracy of the estimated prevalence derived in this study may need to be reassessed. Future research with more rigorous assessments of depression, such as structured interviews by health professionals, could help address the limitations of current findings. Third, the NHANES data did not record the start and end times of antidepressant treatment, nor the use of non-pharmacological treatments, thus partially limiting our study power. Finally, the timeframe of this study did not include the COVID-19 pandemic, which negatively affected people’s mental health and increased the global prevalence of depression by a massive 25% (65). However, despite these shortcomings, to our knowledge, this is the first study to estimate the prevalence of depression and antidepressant treatment in CVD patients using a well-validated dataset representative of the national United States population.

## Conclusion

We elucidated that longitudinal trends in the prevalence of depression among CVD patients in the United States have been stable over the past decade, but was significantly higher in women and those with sleep disorders. Despite the availability of safe and effective treatments, treatment of patients with comorbid depression and CVD remained inadequate. Our findings will provide a reference for future promotion of individualized clinical and pharmacological interventions for high-risk patients.

## Data availability statement

Publicly available datasets were analyzed in this study. This data can be found here: https://www.cdc.gov/nchs/nhanes/.

## Ethics statement

Ethical review and approval was not required for the study on human participants in accordance with the local legislation and institutional requirements. Written informed consent for participation was not required for this study in accordance with the national legislation and the institutional requirements.

## Author contributions

ZF: research idea and study design, statistical analysis, and manuscript drafting. ZF and WT: data acquisition and data analysis and interpretation. ZT: writing–review and editing, supervision, and funding acquisition. All authors contributed important intellectual content during manuscript drafting or revision and read and agreed to the published version of the manuscript.

## Footnotes

1
https://www.icd10data.com/ICD10CM/Codes
